# Fermentation quality, amino acids profile, and microbial communities of whole-plant soybean silage in response to *Lactiplantibacillus plantarum* B90 alone or in combination with functional microbes

**DOI:** 10.3389/fmicb.2024.1458287

**Published:** 2024-11-27

**Authors:** Sijie Jin, Muhammad Tahir, Fuqing Huang, Tianwei Wang, Huangkeyi Li, Weixiong Shi, Yayong Liu, Weichun Liu, Jin Zhong

**Affiliations:** ^1^School of Life Sciences, Yunnan University, Kunming, China; ^2^State Key Laboratory of Microbial Resources, Institute of Microbiology, Chinese Academy of Sciences, Beijing, China; ^3^College of Life Sciences, University of Chinese Academy of Sciences, Beijing, China; ^4^Kulun Banner Agricultural Technology Extension Center, Tongliao, China

**Keywords:** whole plant soybean silage, microbial communities, *Lactiplantibacillus plantarum*, *Bacillus subtilis*, *Saccharomyces cerevisiae*

## Abstract

Promoting the availability of silage with a high protein content on farms can lead to profitable and sustainable ruminant production systems. Whole plant soybean (*Glycine max* L. Merrill, WPS) is a promising high-protein forage material for silage production. In this study, we investigated the fermentation quality, amino acids profile and microbial communities of WPS silage in response to inoculation of lactic acid bacteria (LAB) alone or in combination with non-LAB agents. Before preparing the treatments, the chopped WPS was homogenized thoroughly with 0.3% molasses (0.3 g molasses per 100 g fresh matter). The treatments included CK (sterilized water), LP (*Lactiplantibacillus plantarum* B90), LPBS (LP combined with *Bacillus subtilis* C5B1), and LPSC (LP combined with *Saccharomyces cerevisiae* LO-1), followed by 60 days of fermentation. The inoculants significantly decreased the bacterial diversity and increased the fungal diversity of WPS silage after ensiling. As a result, the contents of lactic acid and acetic acid increased, while the pH value and propionic acid content decreased in the inoculated silages. The amino acids profile was not influenced by inoculants except phenylalanine amino acid, but LP and LPSC silages had substantial greater (*p* < 0.05) relative feed values of 177.89 and 172.77, respectively, compared with other silages. Taken together, the inoculation of LP alone or in combination with BS was more effective in preserving the nutrients of WPS silage and improve fermentation quality.

## Graphical abstract

This study investigated the quality of whole plant soybean (WPS) silage and its microbial communities when inoculated with *Lactiplantibacillus plantarum* B90 (LP) alone or in combination with *Bacillus subtilis* C5B1 (BS) and *Saccharomyces cerevisiae* LO-1 (SC) agents. The results demonstrate that the inoculation of LP alone or in combination with BS is more effective in preserving the nutrients of WPS silage. Moreover, it significantly influences the composition of bacterial and fungal communities, resulting in decreased bacterial diversity and increased fungal diversity. These findings offer valuable insights for the production of protein-rich silage in the future.

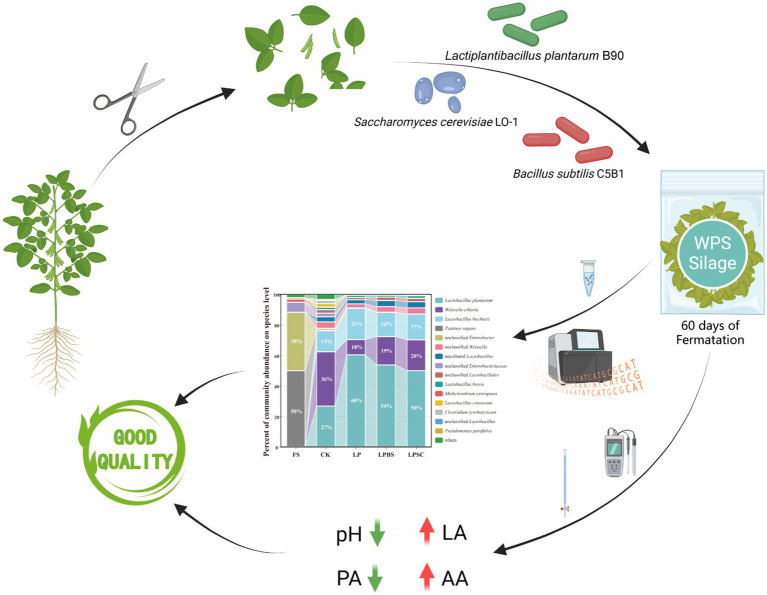

## 1 Introduction

The animal husbandry industry in China has emerged significantly in recent years, owing to the country’s swift economic expansion (Wang et al., 2023a). Despite rapid development of animal husbandry, there is an imbalance between feed demand and supply provided by feed industry. The inadequate availability of high-quality green fodder is the primary limiting factor for further development of animal husbandry (Li et al., 2022a). Recently, ensiling has attracted attention as a forage preservation method to help livestock survive in winters and dry seasons (Yang et al., 2022). Ensiling is a method of storing and processing forage through crushing, compacting, and sealing, which facilitates anaerobic fermentation to produce organic acids, extending the forage’s shelf life and ensuring a year-round supply. Under anaerobic conditions, fermentation drive by lactic acid bacteria (LAB) converts water-soluble carbohydrates (WSC) into organic acids, which are used to lower the pH of silage to inhibit microbial activity and nutrient depletion (Liu et al., 2019). The acidification process is beneficial for the preservation of silages for extended periods with a reduced risk of spoilage. However, unfavorable ensiling conditions can promote clostridial activity, leading to excessive production of ammonia nitrogen (NH_3_-N) and butyric acid (BA), which can result in poor-quality silage.

Promoting the availability of high-protein forages on farms is a profitable and sustainable strategy for ruminant production systems. Legume forages are becoming more popular as a source of protein in dairy farms as an alternative to home-grown protein due to their increased agricultural sustainability benefits such as carbon sequestration and nitrogen fixation, and reduced greenhouse gas emissions by ruminal fermentation and crop N fertilization (Ghizzi et al., 2023). Soybean (*Glycine max* L. Merrill), one of the most valuable oil seed crops, is a promising source of protein for both human and animal diets worldwide (Ni et al., 2017; Dubey et al., 2019). Whole plant soybean (*Glycine max* L. Merrill, WPS) is nutrient rich with high protein and fat contents due to the harvesting of stems, leaves, pods with seeds. The use of WPS as silage for animal feed could be advantageous due to its high protein content, which can help decrease dependence on fluctuating protein prices in the market (Wang et al., 2021). Meanwhile, WPS has a high buffering capacity and low WSC content (Ni et al., 2017), which impairs its silage fermentation profile by increasing the BA content and producing an unpleasant smell, creating a bottleneck problem for its preservation (Carpici, 2016). Therefore, it is of great importance to explore more effective strategies for preservation of WPS in order to fully utilize its potential as a feed source for ruminants.

Various biological additives are applied to enhance fermentation quality of silages, including LAB and molasses. The molasses inoculation generally increases the substrate for LAB proliferation, while LAB generally produce the acids, inhibit protein degradation, and reduce nutrients depletion and improve the fermentation quality by reducing the pH of silage (Ni et al., 2017; Ren et al., 2020). A study has established that 3% addition of molasses substantially improved the fermentation quality (pH < 4.5) and taste of *alfalfa* silage with increased abundance of *Lactobacillus* genus (Luo et al., 2021). Our previous studies have proved that the application of *Lactiplantibacillus plantarum* B90 (formerly *Lactobacillus plantarum* B90) could dominate the silage microbial community, and obviously improve the fermentation quality in many forages, such as soybean silage (Ni et al., 2017), sugarcane top silage (Wang et al., 2020), *Sesbania cannabina* and sweet sorghum mixed silage (Wang et al., 2022). Moreover, some non-LAB agents are also used as additives to perform specific functions during ensiling (Muck et al., 2018). *Bacillus subtilis* could potentially improve the nutritional quality of silages by producing cellulase enzyme, which increase the release of plant cell carbohydrates (Bonaldi et al., 2021). *Bacillus subtilis* can also produce antibiotics, mainly peptides, and antifungal compounds, such as bacillomycin, mycobacillin, and fungistatin (Tabbene et al., 2009), which inhibit the proliferation of undesirable microorganisms, preventing silage from deterioration and mildew (Lara et al., 2016). *Saccharomyces cerevisiae*, a member of yeast family, can help to modulate the immune system of young animals, improve the rumen fermentation, and enhance the nutrient degradability of roughage in the hindgut (Zhou et al., 2019). Studies have reported that population of *Saccharomyces cerevisiae* in corn silage survived during ensiling and increased after feed-out without affecting the silage quality and aerobic stability, when *Saccharomyces cerevisiae* was inoculated at a dose of 10^3^–10^5^ colony forming unit (CFU)/g fresh matter (FM) (Duniere et al., 2015; Xu et al., 2019). Our research group has screened two functional non-LAB strains: *Bacillus subtilis* C5B1 (BS, producing antimicrobial substance), and *Saccharomyces cerevisiae* LO-1 (SC, improving growth performance of cattle, unpublished data). However, how these strains interacts with *Lactiplantibacillus plantarum* B90 (LP) to influence the microbiome structure and fermentation quality of WPS silage is still unknown.

Therefore, current study evaluated the silage quality and microbial communities of WPS silage in response to inoculation of *Lactiplantibacillus plantarum* B90 alone or in combination with BS or SC. We hypothesized that additives synergistically improved silage quality through modulating microbial communities of WPS silage. The results may provide new insights into the regulation mechanism of novel microbial inoculant in silage fermentation, and theoretical support and guidance for future protein-rich silage production.

## 2 Materials and methods

### 2.1 Materials and silage preparation

The WPS (at filling period) was collected from Tongliao City, Inner Mongolia Autonomous Region, China (122°24 E′, 43°65 N′) on September 23, 2020. The WPS was chopped into a particle size of 2 cm using a crop chopper. The chopped WPS was thoroughly homogenized with 0.3% molasses (0.3 g molasses per 100 g fresh matter), then following treatments were applied: CK, sterilized water; LP (*Lactiplantibacillus plantarum* B90); LPBS, LP combined with BS (*Bacillus subtilis* C5B1); and LPSC, LP combined with SC (*Saccharomyces cerevisiae* LO-1). The LP and BS inoculants were applied at theoretical levels of 10^6^ CFU/g fresh weight (FW), while SC inoculant was applied at a theoretical level of 5 × 10^4^ CFU/g FW. An equal amount of sterilized water according to microbial inoculants was prepared for CK group. After spraying of prepared inoculants onto the chopped WPS, about 500 g well-mixed WPS was packed into polyethylene bags and vacuum sealed. The silage bags were kept at temperature around 20–30°C for 60 days (d) to evaluate the fermentation quality, amino acid profile, and microbial communities of WPS silage.

### 2.2 Analysis of fermentation quality and chemical composition

The silage bags were opened after 60 d of ensiling, and 10 g of silage samples were homogenized with 90 mL of sterilized water for 20 min. The resulting mixture was filtered through a 0.22 μm filter, and the filtrate was used to measure pH, organic acids such as lactic acid (LA), acetic acid (AA), propionic acid (PA), BA, and NH_3_-N content. The pH was determined using a glass electrode pH meter (pH 213; HANNA; Italy). Organic acid content was analyzed using high-performance liquid chromatography (HPLC) with an ICSep COREGEL-87H column, and a 210 nm UV detector at a temperature of 55°C. The mobile stage was composed of 0.005 M H_2_SO_4_ with a flow rate of 0.6 mL/min. NH_3_-N concentration was analyzed using the ninhydrin colorimetric and phenol-hypochlorite protocols (Broderick and Kang, 1980).

The post-ensiling samples were dried at 65°C for 48 h in a forced-air oven until a constant weight was achieved to determine the dry matter (DM). To analyze the nutritional components, the dried silage samples were ground into a 1.0 mm particle diameter. The WSC content was determined using the anthrone colorimetric method (Owens et al., 1999), while the measurement of crude protein (CP) was performed using the method described by the Association of Official Analytical Chemists (Helrich, 1990). The levels of neutral detergent fiber (NDF) and acid detergent fiber (ADF) were examined according to the method previously described (van Soest et al., 1991).

### 2.3 Cultivable microbial count

The 20 g of silage samples were blended thoroughly with 180 mL of sterilized saline solution (0.85% NaCl) to homogenize the solution. The homogenized solution was filtered with a single-layer sterilized gauze and then subjected to continuous dilution from 10^0^ to 10^−6^. The filtrate was subsequently inoculated on MRS and PDA (Land Bridge, Beijing, China) under sterile conditions to determine the population of LAB and yeasts, respectively. The MRS plates were incubated under anaerobic conditions at 37°C for 48 h, while the PDA plates were incubated at 25°C for 4 d to estimate the colony count. The microbial populations were expressed as CFU/g of FM and then transformed logarithmically.

### 2.4 Relative feed value

The relative feed value (RFV) was estimated by digestible dry matter (DDM) and dry matter intake (DMI) according to following formula:


$$DDM\;\left(\%DM\right)=88.90-0.779\times ADF\left(\%DM\right)$$

$$DMI\;\left(\%\text{Body}\;\text{weight}\right)=120/NDF\left(\%DM\right)$$

$$RFV=\left(DDM\times DMI\right)/1.29$$

### 2.5 Amino acids profile analysis

The amino acids contents of samples were analyzed by following previously reported method (Tiwari and Jha, 2017). Briefly, the HPLC system (UltiMate 3,000; Thermo Scientific, Waltham, MA, USA) coupled with a fluorescence detector with precolumn derivatization was used to separate and quantify amino acids in silage samples using fluoraldehyde as the reagent (Sedgwick et al., 1991). Prior to injection, the samples were hydrolyzed with 6 mol/L HCl for 24 h at 110°C, except for cysteine, methionine, and tryptophan. An internal standard of *β*-amino-n-butyric acid and ethanol amine mixture was used for analysis. Cysteine content was determined as cysteic acid, methionine content as methionine sulfone after oxidation with performic acid before hydrolysis with 6 mol/L HCl.

### 2.6 DNA extraction, amplification, and sequencing analysis

The DNA extraction was performed according to previously reported method (Li et al., 2022a). The full-length bacterial 16S rRNA genes were amplified by PCR with primers 817F (5′-TTAGCATGGAATAATRRAATAGGA-3′) and 1196R (5′-TCTGGACCTGGTGAGTTTCC-3′), and the fungal internal transcribed spacer (ITS) was amplified with primers ITS1F (5′-CTTGGTCATTTAGAGGAAGTAA-3′) and ITS2R (5′-GCTGCGTTCTTCATCGATGC-3′) primers. The PCR products were analyzed on a 2% agarose gel by electrophoresis. Qualified PCR products were purified using magnetic beads and quantified by enzyme labeling. The purified samples were then mixed equally based on PCR product concentration and loaded onto a 2% agarose gel for detection using glycogel electrophoresis. Target bands were recovered using a gel recovery kit. To ensure PCR accuracy, each sample was set up in three groups for the reaction. The PCR products were sequenced using the Illumina MiSeq platform (Shanghai Majorbio Biopharm Technology Co. Ltd.) with paired terminal read (2 × 300 bp) and standard protocols. Barcodes and primers were removed to obtain high-quality sequencing. Sequences less than 200 bp with a maxhomo*p* value greater than 10 were filtered using Mothur (v.1.34.4). Chimeras were checked in *de novo* mode by USEARCH 8.0 (Edgar, 2013) and remaining sequences were used for downstream analysis. The operational taxonomic units (OTUs) at a 97% similarity level were clustered using QIIME (v1.8.0). Microbial species annotation of OTUs was performed using the SILVA 138 database and UNITE 8.0 database. We have updated the names of *Lactobacillus plantarum*, *Lactobacillus buchneri*, *Lactobacillus brevis* to *Lactiplantibacillus plantarum*, *Lentilactobacillus buchneri* and *Levilactobacillus brevis*, respectively, according to the new taxonomic system in the text. The resulting OTUs file was used for calculating rarefaction [R (v.22)] and alpha diversity [Mothur (v1.34.4)]. We also used linear discriminant analysis effect size (LEfSe) to identify significant associations between bacterial and fungi taxa in the treatments. LEfSe was performed using the OmicStudio tools at: https://www.omicstudio.cn/tool/.

### 2.7 Statistical analysis

The reported results represent the mean of three replicates. Statistical analysis was conducted using GraphPad Prism (version 8.0.0, San Diego, California, USA). One-way analysis of variance was conducted for multiple groups followed by Duncan’s multiple range test. The spearman correlation coefficients were calculated to determine the relationships between the microbiome and silage quality variables. The correlation coefficients were plotted using the “pheatmap” libraries in R. Statistical significance was declared at a threshold of *p* < 0.05.

## 3 Results

### 3.1 Fermentation profile of WPS silage

**Figure 1:**
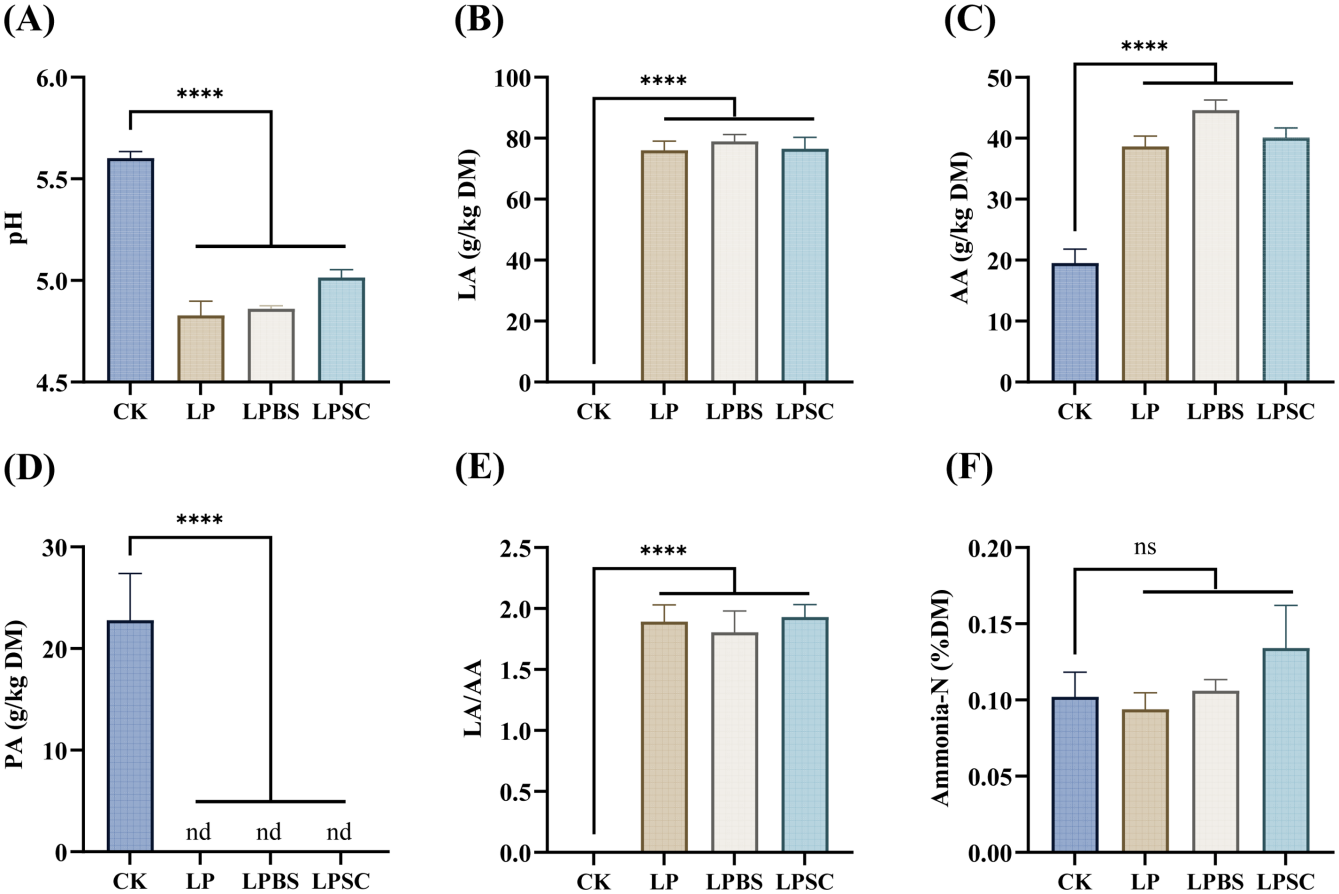
Fermentation characteristics of whole plant soybean silages after 60 days of ensiling. **(A)** For pH; **(B)** for LA; **(C)** for AA; **(D)** for PA; **(E)** for LA/AA; and **(F)** for ammonia-N. CK, sterilized water; LP, *Lactiplantibacillus plantarum* B90; LPBS, LP combined with *Bacillus subtilis* C5B1; LPSC, LP combined with *Saccharomyces cerevisiae* LO-1; ns, non-significance; nd, no detection; ****p < 0.001.

The fermentation profile of WPS silage is presented in Figure 1. The inoculated silages had substantial lower pH values compared with CK silage (*p* < 0.001); specifically, LP silage exhibited lower pH value of 4.83 compared with CK silage (5.60), but it was not substantially different than other inoculated silages (Figure 1A). The inoculated silages had substantial greater LA and AA contents compared with CK silage (*p* < 0.001), but neither LA content nor AA content was substantially different among inoculated groups and were ranged from 76.05–78.94 g/kg DM and 38.66–44.59 g/kg DM, respectively (Figures 1B,C). The PA content was only found in CK silage with value of 22.78 g/kg DM, while it was not found in inoculated silages (Figure 1D). The LA/AA ratios were significantly higher in inoculated silages compared with CK silage (*p* < 0.001), but there was no substantial difference among inoculated silages (Figure 1E). However, NH_3_-N content was not significantly different among silages, but LP silage had numerically lower NH_3_-N content of 0.09 %DM compared with other groups (Figure 1F). Taken together, results presented here highlighted that LP inoculation alone or in combination with BS or SC has similar fermentation quality of WPS silage.

### 3.2 Chemical composition and cultivable microbial counts of WPS silage

The chemical composition and cultivable microbial counts of WPS silage are depicted in Table 1. The inoculated silages had significant greater DM contents and lower DM losses compared with CK silage (*p* < 0.001), but neither DM content nor DM loss was substantially different among inoculated groups and were ranged from 29.60 to 29.93% and 1.80 to 2.44%, respectively. The contents of CP were not influenced by inoculants and were between 22.82 and 23.94 %DM, the contents of WSC decreased with the addition of inoculant and ranged from 0.52 to 0.60 %DM (*p* < 0.05), respectively. The NDF and ADF contents of the LP and LPSC silages were significantly lower by 4.07 and 3.35%, and 4.35 and 3.58%, respectively, compared to the CK silage (*p* < 0.001). The LP and LPSC silages had substantial higher RFV of 177.89 and 172.77, respectively, compared with other silages. Taken together, results suggested that all inoculants were effective in conserving the nutrients of WPS silage, but inoculation of LP alone or in combination with SC may perform better to degrade the digestible fiber. The inoculated silages had significant higher LAB and yeast counts than CK silage (*p* < 0.05), but neither LAB count nor yeast count was substantially different among inoculated silages and were ranged from 9.02 to 9.15 and 8.76 to 8.83 log10 CFU/g FM, respectively.

**Table 1:** Chemical characteristics and cultivable microbial count of whole plant soybean silages after 60 days of ensiling.

Items	Treatments				SEM	Significance
	CK	LP	LPBS	LPSC		*p*-value
Chemical composition						
DM (%)	28.58^b^	29.79^a^	29.60^a^	29.93^a^	0.1375	0.000
DM loss (%)	3.72^a^	1.81^b^	2.45^b^	2.18^b^	0.2031	0.000
CP (%DM)	22.82^a^	23.94^a^	23.24^a^	23.57^a^	0.2095	0.296
NDF (%DM)	38.94^a^	34.87^c^	36.90^b^	35.59^c^	0.3808	0.000
ADF (%DM)	32.89^a^	28.54^c^	30.24^b^	29.31^bc^	0.4071	0.000
NDIP (%DM)	1.71^a^	1.53^b^	1.66^ab^	1.59^ab^	0.0253	0.052
ADIP (%DM)	1.32^a^	1.10^b^	1.21^ab^	1.18^ab^	0.0297	0.047
WSC (%DM)	0.60^a^	0.52^b^	0.55^ab^	0.52^b^	0.0108	0.033
RFV	151.26^c^	177.89^a^	164.77^b^	172.77^a^	2.4679	0.000
Cultivable microbes						
LAB (log 10 CFU/g FM)	8.47^b^	9.15^a^	9.02^a^	9.10^a^	0.0629	0.000
Yeast (log 10 CFU/g FM)	8.50^b^	8.83^a^	8.80^a^	8.76^a^	0.0384	0.001

CK, sterilized water; LP, *Lactiplantibacillus plantarum* B90, LPBS, LP combined with *Bacillus subtilis* C5B1; LPSC, LP combined with *Saccharomyces cerevisiae* LO-1; DM, dry matter; CP, crude protein; NDF, neutral detergent fiber; ADF, acid detergent fiber; NDIP, neutral detergent insoluble protein ADIP, acid detergent insoluble protein; WSC, water soluble carbohydrates; RFV, relative feed value; LAB, lactic acid bacteria; CFU, colony forming unit; FM, fresh matter. Different small letters indicate the significant difference among treatments. The significance was employed at probability level 0.05.

### 3.3 Amino acids profile of WPS silage

The amino acids composition of WPS silage is presented in Table 2. There were no substantial differences for amino acids contents except phenylalanine among all silages, and were ranged from 0.12 to 2.02 %DM. However, the content of phenylalanine amino acid was substantial higher in LPBS silage with quantity of 0.94 %DM compared with other silages (*p* < 0.01). Taken together, the inoculants did not influence the amino acids composition except phenylalanine of WPS silage.

**Table 2:** Amino acids profile of whole plant soybean silage.

Amino acids (%DM)	Treatments				SEM	Significance
	CK	LP	LPBS	LPSC		*p-*value
Aspartic acid	1.39	1.56	1.81	1.48	0.0972	0.506
Threonine	0.38	0.45	0.47	0.41	0.0268	0.702
Serine	0.38	0.32	0.36	0.29	0.0211	0.468
Glutamic acid	2.02	1.65	1.89	1.53	0.1097	0.418
Proline	1.06	0.94	1.03	0.88	0.0544	0.707
Glycine	1.09	0.89	0.92	0.91	0.0555	0.630
Alanine	1.00	0.82	0.91	0.78	0.0488	0.458
Cystine	0.26	0.08	0.19	0.15	0.0309	0.218
Valine	0.85	0.72	0.82	0.69	0.0435	0.579
Methionine	0.12	0.09	0.11	0.09	0.0078	0.650
Isoleucine	0.69	0.61	0.70	0.57	0.0366	0.570
Leucine	1.15	0.97	1.12	0.93	0.0594	0.547
Tyrosine	0.25	0.22	0.22	0.20	0.0129	0.745
Phenylalanine	0.86^ab^	0.62^bc^	0.94^a^	0.54^c^	0.0616	0.034
Lysine	0.90	0.83	0.94	0.78	0.0485	0.729
Histidine	0.35	0.31	0.35	0.30	0.0167	0.731
Arginine	0.43	0.35	0.41	0.32	0.0232	0.445

CK, sterilized water; LP, *Lactiplantibacillus plantarum* B90, LPBS, LP combined with *Bacillus subtilis* C5B1; LPSC, LP combined with *Saccharomyces cerevisiae* LO-1. Data is the mean of three replicates. Different small letters indicate the significant difference among treatments. The significance was employed at probability level 0.05.

### 3.4 Bacterial community of fresh and fermented WPS

The alpha diversity analysis revealed a decrease of the bacterial biodiversity in WPS silage when it was inoculated with LP alone or with in combination with BS or LC compared with CK silage (Supplementary Figures S1A,B). More specifically, the greater decrease in bacterial biodiversity was seen in LP silage compared with other inoculated silages. The principal coordinate’s analysis (PCoA) based on OTU level was applied to identify the factors that influence the variations in microbiome of WPS silage (beta diversity). The results showed a substantial bacterial species succession dynamic when inoculants were applied, while they were indistinguishable among silage inoculated with LP alone or with in combination with BS or LC (Supplementary Figure S1C).

**Figure 2:**
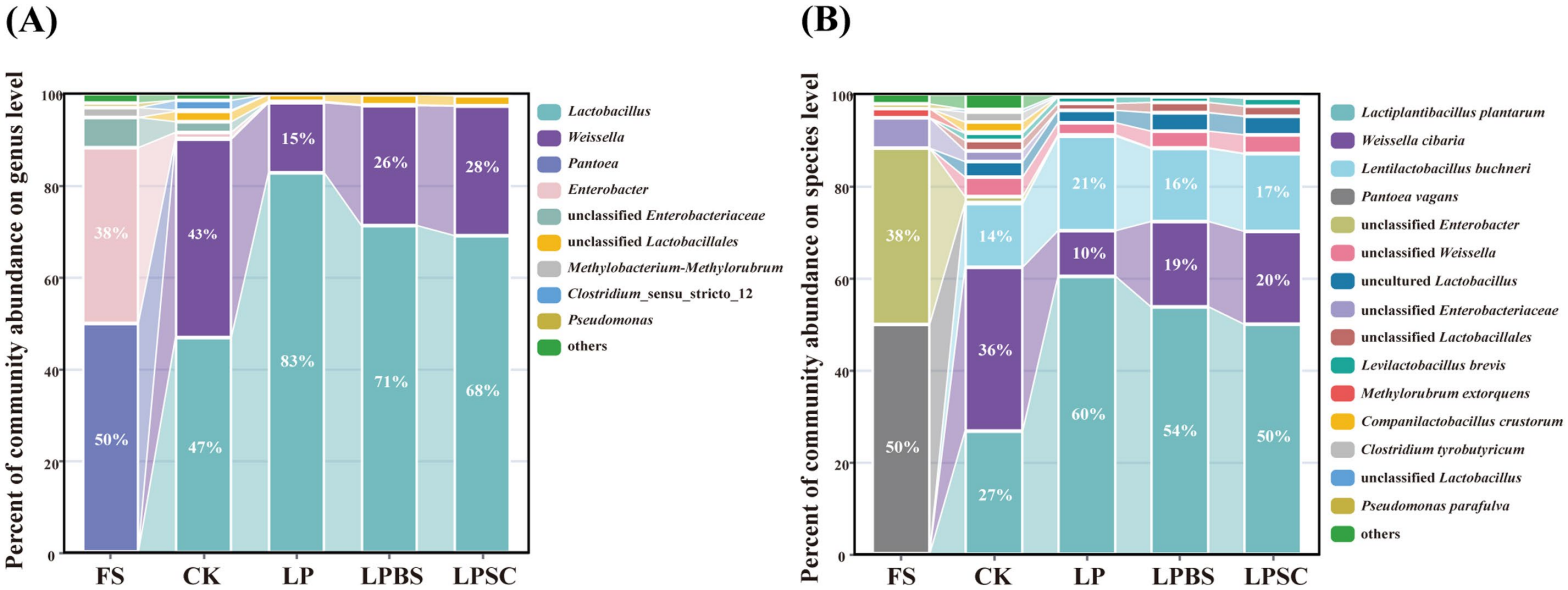
Microbial community of whole plant soybean silages at genus **(A)** and species **(B)** levels after 60 days of ensiling. CK, sterilized water; LP, *Lactiplantibacillus plantarum* B90; LPBS, LP combined with *Bacillus subtilis* C5B1; LPSC, LP combined with *Saccharomyces cerevisiae* LO-1.

The relative abundance of bacterial communities in WPS before and after ensiling are shown in Figures 2A,B. At genus level (Figure 2A), epiphytic microflora of fresh WPS was mainly comprised of unwanted bacteria for ensiling such as *Pantoea* (50%) and *Enterobacter* (38%). After 60 d of fermentation, *Lactobacillus* and *Weissella* were dominant genera in CK silage, accounting relative abundances of 47 and 43%, respectively. The inoculants substantially increased the relative abundance of *Lactobacillus* and decreased the relative abundance of *Weissella* in WPS silage. The LP silage had the greater relative abundance of *Lactobacillus* (83%) and lower relative abundance of *Weissella* (15%) compared with other inoculated silages. The relative abundances of *Lactobacillus* and *Weissella* in LPBS and LPSC silages were 71 and 26%, and 68 and 28%, respectively. At specie level (Figure 2B), the *Pantoea vagans* (50%) and unclassified *Enterobacter* (38%) were dominant species in fresh WPS. After 60 d of fermentation, the *Weissella cibaria*, *Lactiplantibacillus plantarum* and *Lentilactobacillus buchneri* were dominant species in CK silage comprising the relative abundances of 36, 27, and 14%, respectively. The inoculants substantially increased the relative abundance of *Lactiplantibacillus plantarum* and decreased the relative abundance of *Weissella cibaria* in WPS silage (Supplementary Figure S3). The relative abundances of *Lactiplantibacillus plantarum* and *Weissella cibaria* in LP, LPBS and LPSC silages were 60, 54, and 50%, and 10, 19, and 20%, respectively (Figure 2B). Taken together, inoculants significantly influenced the bacterial community of WPS silage, particularly increased the relative abundance of beneficial *Lactiplantibacillus plantarum* in WPS silage.

The LEfSe analysis revealed that the LP, LPBS, and LPSC groups had higher *Lactiplantibacillus plantarum* enrichment, while the CK group had higher enrichment of *Leuconostocaceae*, *Weissella* and *Clostridiaceae* suggesting that this different microbial composition contributed to the difference in fermentation quality (Supplementary Figures S5A–C). The LP group had higher *Lactobacillus* enrichment compared to the LPBS and LPSC groups, while the LPBS and LPSC groups had higher *Weissella* enrichment, probably because the activity of the added BS and SC in the early fermentation phase which affected the microbial composition of the silage, as well as the growth of the LP (Supplementary Figures S5D,E). The LPBS and LPSC groups analyses showed that there were also differences in microbial species between them, with LPBS having a higher enrichment of *Enterobacterales*, *Gammaproteobacteria* and LPSC having a higher enrichment of *Sphingomonadales* (Supplementary Figure S5F).

### 3.5 Fungal community in fresh and fermented WPS

The alpha diversity analysis indicated an increase in fungal biodiversity of WPS silage when it was inoculated with LP alone or in combination with BS or LC compared with CK silage (Supplementary Figures S2A,B). The PCoA based on OTU level was applied to identify the factors that that influence the variations in fungal community of WPS silage (beta diversity). The results suggested a substantial fungal species succession change when inoculants were applied, while they were indistinguishable among inoculated silages (Supplementary Figure S2C).

**Figure 3:**
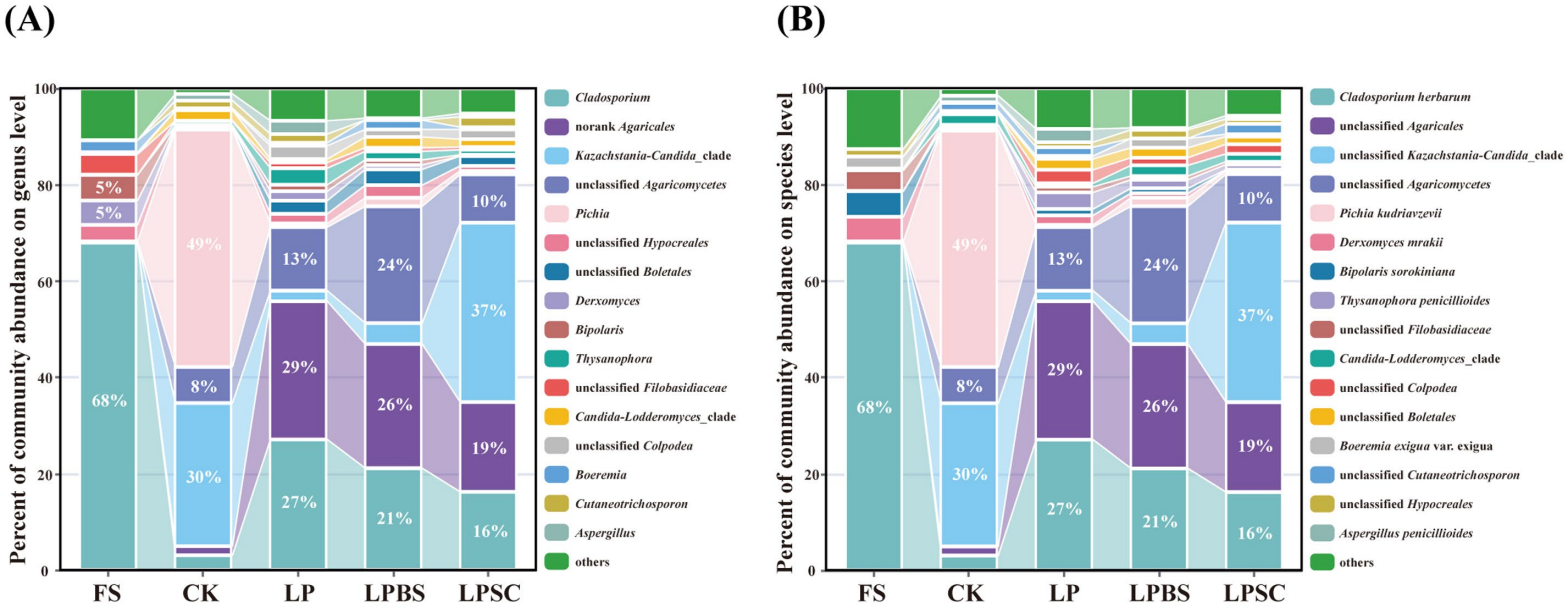
Fungal community of whole plant soybean silages at genus **(A)** and species **(B)** levels after 60 days of ensiling. CK, sterilized water; LP, *Lactiplantibacillus plantarum* B90; LPBS, LP combined with *Bacillus subtilis* C5B1; LPSC, LP combined with *Saccharomyces cerevisiae* LO-1.

The relative abundance of fungal communities in WPS before and after ensiling are shown in Figures 3A,B. At genus level (Figure 3A), epiphytic fungal microflora of fresh WPS was mainly comprised of *Cladosporium*, *Derxomyces*, and *Bipolaris*, accounting relative abundances of 68, 5, and 5%, respectively. After 60 d of fermentation, *Pichia*, *Kazachstania-Candida*_clade, and unclassified *Agaricomycetes* were most prevalent genera in CK silage comprising the relative abundances of 49, 30, and 8%, respectively. The inoculants substantially influenced the diversity of fungal microflora in WPS silage after 60 d of fermentation. The *Cladosporium*, norank *Agaricales*, and unclassified *Agaricomycetes* were dominant genera in LP and LPBS silages accounting the relative abundances of 27 and 21%, 29 and 26%, and 13 and 24%, respectively. In LPSC silage, the main fungal genera were comprised of *Kazachstania-Candida*_clade, norank *Agaricales*, *Cladosporium* and unclassified *Agaricomycetes* accounting the relative abundances of 37, 19, 16, and 10%, respectively. At specie level (Figure 3B), epiphytic fungal microflora of fresh WPS was mainly comprised of *Cladosporium herbarum* (68%). After 60 d of fermentation, *Pichia kudriavzevii* (49%), unclassified *Kazachstania-Candida*_clade (30%), and unclassified *Agaricomycetes* (8%) were most abundant fungal species in CK silage. The inoculants substantially influenced the fungal species and decreased the abundance of *Pichia kudriavzevii* (Supplementary Figure S4); and *Cladosporium herbarum*, unclassified *Agaricales*, and unclassified *Agaricomycetes* were most dominant species in LP and LPBS silages comprising the relative abundances of 27 and 21%, 29 and 26%, and 13 and 24%, respectively (Figure 3B). However, unclassified *Kazachstania-Candida*_clade (37%), unclassified *Agaricales* (19%), *Cladosporium herbarum* (16%), and unclassified *Agaricomycetes* (10%) were most prevalent fungal species in LPSC silage. Taken together, inoculants substantially increased the fungal diversity and substantially decreased the relative abundance of *Pichia kudriavzevii* in WPS silage.

The LEfSe analysis revealed that both the LP and LPBS groups had higher enrichment of *Basidiomycota* and *Agaricomycetes* compared to the CK group. In contrast, the LPSC group exhibited only an increased enrichment of *Agaricomycetes*. Compared to other groups, the CK group showed higher levels of *Saccharomycetes* and *Pichia kudriavzevii* (Supplementary Figures S6A–C), indicating a distinct difference in fungal composition that may influence the aerobic stability of WPS silage. The LPBS group demonstrated a greater enrichment of *Agaricomycetes*, while the LPSC group exhibited higher levels of *Dothideomycetes* and *Trichomeriaceae* enrichment compared to LP group, suggesting that the addition of SC significantly altered the fungal community composition in WPS silage (Supplementary Figures S6D,E).

### 3.6 Correlations between microbiome and fermentation products of WPS silage

**Figure 4:**
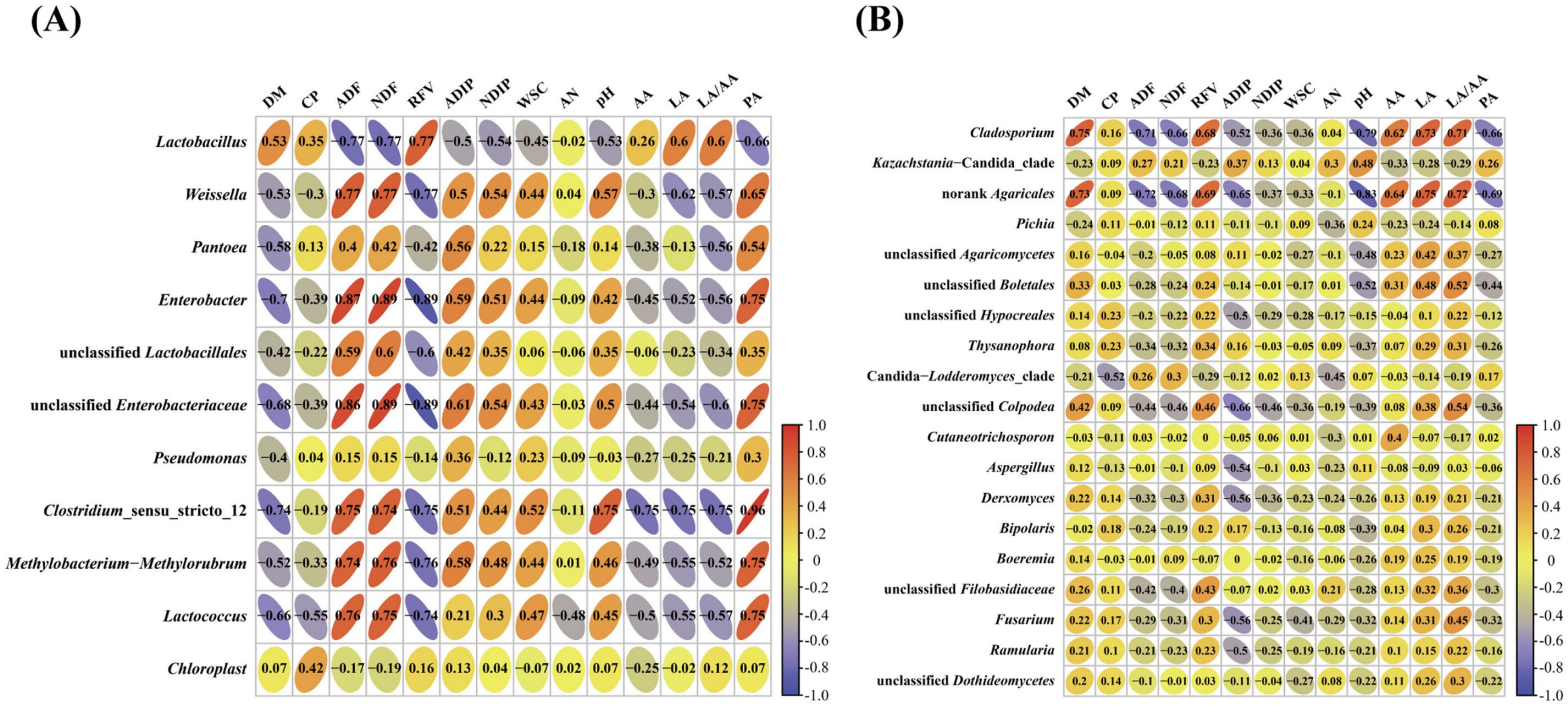
Associations between fermentation products and bacterial **(A)** and fungal **(B)** communities. DM, dry matter; CP, crude protein; NDF, neutral detergent fiber; ADF, acid detergent fiber; WSC, water soluble carbohydrates; RFV, relative feed value; AN, ammonia-N; LA, lactic acid; AA, acetic acid; PA, propionic acid.

To further characterize the effects of bacterial and fungal species on fermentation quality, the correlation analysis between bacterial and fungal genera and fermentation quality of silages was investigated (Figure 4). The results indicated that *Lactobacillus* was positively correlated with RFV, LA and LA/AA and negatively correlated with ADF and NDF, whereas *Weissella*, *Enterobacter*, *Clostridium*_sensu_stricto_12, unclassified *Enterobacteriaceae* and *Lactococcus* were positively correlated with ADF and NDF and negatively correlated with LA (Figure 4A). The *Cladosporium* and norank *Agaricales* were negatively correlated with NDF, ADF, pH and PA, and positively correlated with DM, RFV, LA, AA and LA/AA, whereas *Kazachstania-Candida*_clade was positively correlated with pH (Figure 4B).

## 4 Discussion

### 4.1 LAB and functional strains enhance WPS silage fermentation and nutrient preservation

Soybean is widely planted worldwide as for both human and ruminant’s feed. However, its low WSC content and high buffer capacity made it difficult to be ensiled. Inoculating silage with LAB and molasses was found to ensure good fermentation quality because LAB can efficiently transform WSC into LA and reduce silage pH to inhibit the growth of undesirable microorganisms at the initial stage of ensiling (Li et al., 2022b). Moreover, some non-LAB inoculants may have the ability to secrete cellulose-related enzymes that help in hydrolyzing the fiber structure of forages and release more fermentable sugar during ensiling (Bai et al., 2020), whereas some species have potential to improve the rumen fermentation and can be applied as a direct-fed microbial strain at ensiling (Zhu et al., 2017). Therefore, in this study, we explored the addition of lactic acid-producing bacteria (LP) with or without non-LAB agents (namely BS and SC) and molasses as additives before ensiling. The goal was to enhance the fermentation of WPS silage and tackle the challenges related to the challenging ensiling of WPS.

The silages treated with LP, LPBS, and LPSC had higher LA and AA contents compared with CK silage, whereas no significance difference was found among inoculated silages. Previous studies have shown that LAB strains can decline pH value by accelerating WSC transformation into lactic and acetic acids (Ding et al., 2019; Zhang et al., 2021). Silages treated with inoculants were found to have the lowest pH values compared to CK silage, whereas no substantial differences in pH values were found between LP, LPBS, and LPSC silages after 60 d of ensiling. The most reasonable account was that *Lactiplantibacillus plantarum* dominated the silage bacterial community during the fermentation (Keshri et al., 2018). In addition, a study has shown that combined inoculation of LP and BS raised the LA content and decreased the pH of corn silage (Lara et al., 2018), whereas some studies have reported that SC inoculation at a dose of 10^3^–10^5^ CFU/g of fresh forage did not alter the nutritional quality, fermentation traits, and *Lactobacillus* populations of corn silage and survived during ensiling (Duniere et al., 2015; Xu et al., 2019). Therefore, it can be speculated that the antimicrobial peptide producing ability of BS that could accelerate the growth of the homofermentative LAB and accurate dose of SC inoculation with LP did not restrict each other’s effect but benefited fermentation by increasing LA and AA concentrations compared to those in the CK silage. Generally, PA is produced from secondary fermentation of *Clostridia* by consuming LA. The PA was not found in inoculated silages compared to CK silage, indicating that application of microbial inoculants inhibited secondary fermentation thereby preserving more nutrients excellently (Li et al., 2020).

The lower DM losses in inoculated silages compared with CK silage further proved that additives are favorable to WPS ensilage in the present study. After 60 d of fermentation, inoculated silages LP, LPBS, and LPSC had remarkably higher DM concentrations compared with CK silage, indicating that LAB inhibited the spoilage microbes and their fermentation activities by reducing pH of silage which agrees with previous study (Zhang et al., 2023a). The consumption rate of WSC is associated with the extent of fermentation (Long et al., 2022). However, the WSC content was not affected by inoculants and there was no substantial difference among inoculated silages and CK silage. This could be attributed to the 0.3% molasses addition in this study which provided sufficient fermentable substrates to LAB to carry on the fermentation. Proteolysis mainly resulted from plant proteases, but it could be inhibited when the pH has declined (Tahir et al., 2023). The addition of inoculants in the silages did not result in a reduction of NH_3_-N content after 60 d of ensiling, which can be further confirmed by the CP content in all silages. This result might be attributed to the presence of aerobic bacteria and clostridia in all silages (Kung and Shaver, 2001). The substantial degradation of NDF and ADF contents was found in LP and LPSC silages compared with CK silage which could be the co-action of various enzymes produced by the LAB to hydrolyze more digestible cell wall fractions. Numerous studies have found that inoculation with LAB promoted the degradation of lignocellulose (Li et al., 2022a; Tahir et al., 2023). In this study, the degradation of NDF and ADF contents in LPBS silage was lower than other inoculated silages suggesting that combined inoculation of LP and BS restricted each other effect on cell wall degradation. Moreover, the inoculants did not influence the amino acids profile of WPS silage suggesting that inoculants were not able to efficiently break down proteins into amino acids, leading to minimal impact on the amino acid composition.

### 4.2 Inoculants influence bacterial community and enhance WPS silage quality

The fermentation quality of silages with or without additives depends on the bacterial composition and changes during the ensiling time (Xu et al., 2021). Bacterial alpha diversity of WPS decreased after fermentation, and silage with inoculants had lower bacterial alpha diversity after fermentation, which could be due to the dramatic decrease in pH value. The acidic anaerobic environment after ensiling led to the modification of the bacterial community where most of the epiphytic bacteria disappeared due to their inability to adapt to low pH (Zhang et al., 2023b). The presence of *Pantoea* in silage is generally considered undesirable because it competes with LAB for sugars (Jiang et al., 2020), while *Enterobacter* has the potential to ferment LA into AA and other products, leading to significant nutrition loss (Ni et al., 2017). In the present study, the inoculants substantially decreased the relative abundance of *Enterobacter*, *Pantoea* and other epiphytic bacteria, and increased the relative abundance of *Lactobacillus* after ensiling. This could be attributed to the adaptability as well as the rapid growth and multiplication of *Lactobacillus* species which produces higher LA that swiftly declined the pH to inhibit the growth of spoilage microorganisms during the fermentation (Yang et al., 2019; Bai et al., 2020). The *Lactobacillus* genus is known to regulate the fermentation process in anaerobic conditions and could grow vigorously during fermentation owing to its high acid resistance (Muck et al., 2018). In the present study, the relative abundance of *Lactobacillus* was greater, while the relative abundance of *Weissella* was lower in LP silage; however, the relative abundance of *Lactobacillus* decreased and the relative abundance of *Weissella* increased when WPS was inoculated with LP in combination with BS or SC in agreement with the results of pH and LA. This highlights that combined inoculation of BS or SC with LP slightly weakened the growth of *Lactobacillus*, which might be related to their different metabolic functions leading to competition among each other. The *Weissella* is typically considered an early colonizer, but is eventually surpassed by acid-resistant *Lactobacillus* due to the pH decline during fermentation (Graf et al., 2016), which explains its higher relative abundances in silages with greater pH value in the present study. Moreover, the *Weissella cibaria* was enriched bacterial specie in the CK silage; and most possible explanation lies in the high pH value after 60 d of silage fermentation. As expected, inoculants increased the relative abundance of *Lactiplantibacillus plantarum* specie in the treated silages compared with the control silage. This result suggested that inoculation of LP alone or in combination with BS or SC could accelerate the growth of *Lactobacillus* species by providing conducive environment for their proliferation resulting in better silage quality.

### 4.3 Inoculants altered the fungal community which may potentially enhance the aerobic stability of WPS silage

The fungal community in silage is complex, comprising various species, including yeasts, molds and filamentous fungi. In the present study, fungal alpha diversity of WPS increased after ensiling, and silage with inoculants had higher fungal alpha diversity compared with control silage after fermentation. Likewise, a study has found that the fungal richness and diversity in barley silage inoculated with LAB was higher than that of untreated silage during ensiling (Liu et al., 2019). The *Cladosporium* was the main fungal genus in the fresh WPS, but its growth was substantially inhibited after ensiling, suggesting the need for oxygen by latter. *Cladosporium* produces mycotoxins during their growth by infections of forages, posing a significant risk to livestock health during ensiling and grazing (Huang et al., 2022). In the present study, the fungal community was mainly represented by *Pichia*, *Kazachstania-Candida*_clade, unclassified *Agaricomycetes*, *Cladosporium*, and norank *Agaricales* genera after ensiling, supported by previous studies (Xu et al., 2019; Wang et al., 2020). The *Pichia* and *Kazachstania-Candida*_clade were most prevalent fungal genera in CK silage and generally these considered as spoilage microbes during aerobic exposure of silage (Zhang et al., 2019). The presence of these spoilage microbes in CK silage might be related to its greater pH value which was conducive for the growth of these fungi (Wang et al., 2022). Moreover, the addition of inoculants substantially changed the fugal community and *Cladosporium*, unclassified *Agaricomycetes*, and norank *Agaricales* became the dominant genera in inoculated silages, suggesting that these fungi could proliferate in greater acidic environment. Meanwhile, it was quite fascinating to found the substantial higher relative abundance of *Kazachstania-Candida*_clade genus in LPSC silage, which might be related to its lower acidic environment compared to other treated silages and inoculation of SC which provided stable environment for its growth. This result suggests that addition of LP with SC may not reduce aerobic deterioration caused by the post-spoilage fungal growth such as *Kazachstania-Candida*_clade and *Cladosporium* (Wang et al., 2020).

The main fungal specie observed in fresh WPS was *Cladosporium herbarum*. The *Cladosporium herbarum* could grow at low temperatures, and usually associated with foods spoilage and discoloration (Bullerman, 2003). The *Pichia kudriavzevii* and unclassified *Kazachstania-Candida*_clade were dominant fungal species in CK silage which might be related to its higher pH value; and these species have been involved in silage corruption during aerobic exposure (Santos, 2011; Carvalho et al., 2017). Meanwhile, the addition of LP alone or in combination with BS substantially inhibited the growth of *Pichia kudriavzevii* and unclassified *Kazachstania-Candida*_clade, and increased the relative abundances of unclassified *Agaricales*, *Cladosporium herbarum*, and unclassified *Agaricomycetes* fungal species after ensiling which could be attributed to lower pH values of LP and LPBS silages. This result suggests that addition of LP alone or in combination with BS could improve the aerobic stability of WPS silage by inhibiting the growth of spoilage fungal species. However, unclassified *Kazachstania-Candida*_clade and unclassified *Agaricales* were the most abundant fungal species in LPSC silage that could be directly related to the addition of SC which provided conducive environment for their growth. Moreover, the population of SC did not survive after ensiling of WPS when LPSC was inoculated and the reason for this is unknown. This phenomenon may be related to the aerobic conditions required for SC survival. At the beginning of fermentation, the residual oxygen in the silage likely provided favorable conditions for SC, allowing it to survive temporarily. During this early stage, SC may have contributed to a rise in the silage temperature, influencing the microbial composition. As the residual oxygen was consumed, LAB and other fast-growing anaerobic microbes began to dominate the fermentation. Due to the lack of a suitable growth environment, SC was eventually decomposed. However, this hypothesis requires further validation through subsequent experiments. This result highlights that addition of LP with SC may not improve the aerobic stability of WPS after exposure to air likewise to previous study (Xu et al., 2019), as unclassified *Kazachstania-Candida*_clade was abundant. However, it has been reported that the relative quantitative analyzes cannot reflect the true absolute abundance of microorganism groups in a sample. For instance, the increase in the relative abundance of a certain group of microorganisms may not reflect the increase of its absolute abundance. Instead, it may be linked to the decrease in the absolute abundance of other microorganisms (Yang et al., 2021).

### 4.4 Interaction of multiple microorganisms and metabolites influences fermentation quality of WPS silage

Silage fermentation is a very complex biological process involving a variety of microorganisms and biochemical reactions, and produces many different metabolites during ensiling (Wang et al., 2023b; Xin et al., 2023). Understanding the relationship between metabolites with fermentation bacteria and fungi can provide better insight into the fermentation mechanism of silage. *Lactobacillus* is the core bacteria to produce LA during ensiling process and determine the silage quality. Like previous studies (Guan et al., 2018; Xia et al., 2022; Tahir et al., 2023), the current study also reported that concentration of LA is positively correlated with *Lactobacillus*. The unclassified *Enterobacteriaceae* and *Lactococcus* had positive associations with ADF, NDF and PA, while negative correlations with DM, RFV, and LA, highlighting that these bacteria are involved in silage corruption. The *Cladosporium* and norank *Agaricales* were positively correlated with LA and negatively correlated with pH, highlighting that these species are beneficial to preserve the silage nutrients. However, a study has reported that *Cladosporium* can produce mycotoxins posing a significant health to livestock (Huang et al., 2022), therefore, it is important to manage and minimize the presence of *Cladosporium* in silage through proper harvesting, storage, and management practices. *Kazachstania* might have a strong tolerance to LA and be a yeast species that is crucially involved in initiating the aerobic deterioration of silage with a relatively low pH and AA content which is in line with the result of present study as a positive association between *Kazachstania-Candida*_clade and pH was observed.

## 5 Conclusion

The addition of LP alone or in combination with BS or SC substantially improved the WPS silage quality by increasing the LA and AA contents and decreasing the pH value and the PA content. The inoculants had no impact on the amino acid profile of WPS silage. However, it is also substantially influenced the bacterial and fungal communities, resulting in reduced bacterial diversity and increased fungal diversity. The *Lactobacillus* was the most prevalent bacterial genus in the inoculated silages, while *Cladosporium*, norank *Agaricales*, and unclassified *Agaricomycetes* were dominant fungal genera after 60 d of ensiling. Moreover, the bacterial and fungal communities were substantially correlated with fermentation characteristics. Taken together, the inoculation of LP alone or in combination with BS was more effective in preserving the nutrients of WPS silage which may provide theoretical support and guidance for future protein rich silage production.

## Data Availability

The datasets presented in this study can be found in online repositories. The names of the repository/repositories and accession number(s) can be found at: https://www.ncbi.nlm.nih.gov/, PRJNA1068694.
