# Supplementary material for: Fermentation quality, amino acids profile, and microbial communities of whole-plant soybean silage in response to Lactiplantibacillus plantarum B90 alone or in combination with functional microbes
Source: Front Microbiol. 2024 Nov 27;15:1458287. doi: 10.3389/fmicb.2024.1458287 (PMC11631902; doi:10.3389/fmicb.2024.1458287)
Supplement: Supplementary file 1 [file Supplementary_file_1.pdf]

Supplementary Material

Fermentation quality, amino acids profile and microbial communities of whole-plant soybean silage in response to *Lactiplantibacillus plantarum* B90 alone or in combination with functional microbes

Sijie Jin†, Muhammad Tahir†, Fuqing Huang, Tianwei Wang*, Huangkeyi Li, Weixiong Shi, Yayong Liu, Weichun Liu, Jin Zhong*

*** Correspondence:** Tianwei Wang: wangtw@im.ac.cn; Jin Zhong: zhongj@im.ac.cn.

1 Supplementary Figures

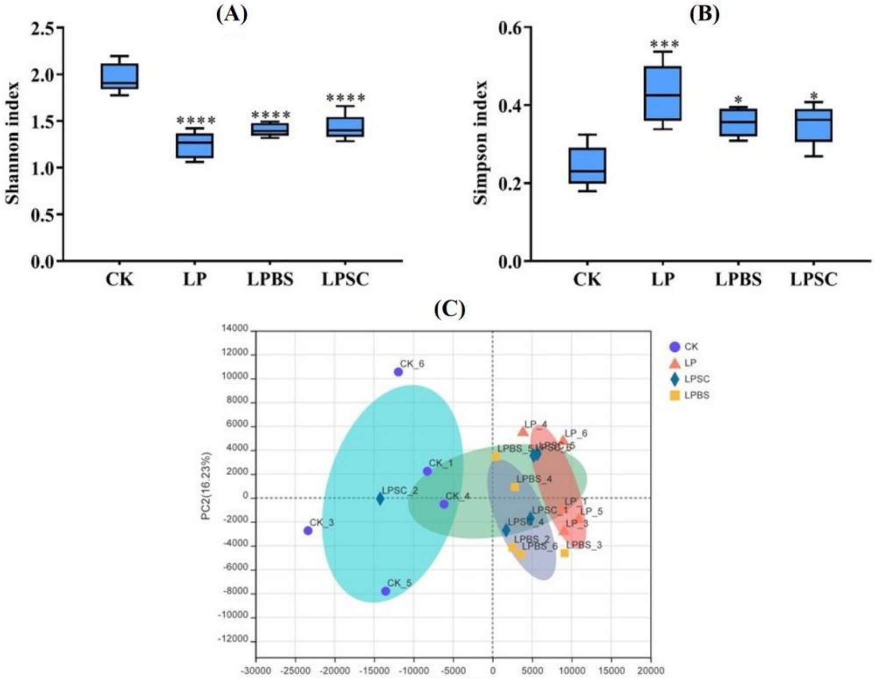

Supplementary Figure 1. Alpha (Shannon and Simpson indices) and beta (PCA) diversities analysis of bacterial community of whole plant soybean silage after 60 days of ensiling. A for Shannon index; B for Simpson index; C for PCA. CK, sterilized water; LP, *Lactiplantibacillus plantarum* B90; LPBS, LP combined with *Bacillus subtilis* C5B1; LPSC, LP combined with *Saccharomyces cerevisiae* LO-1.

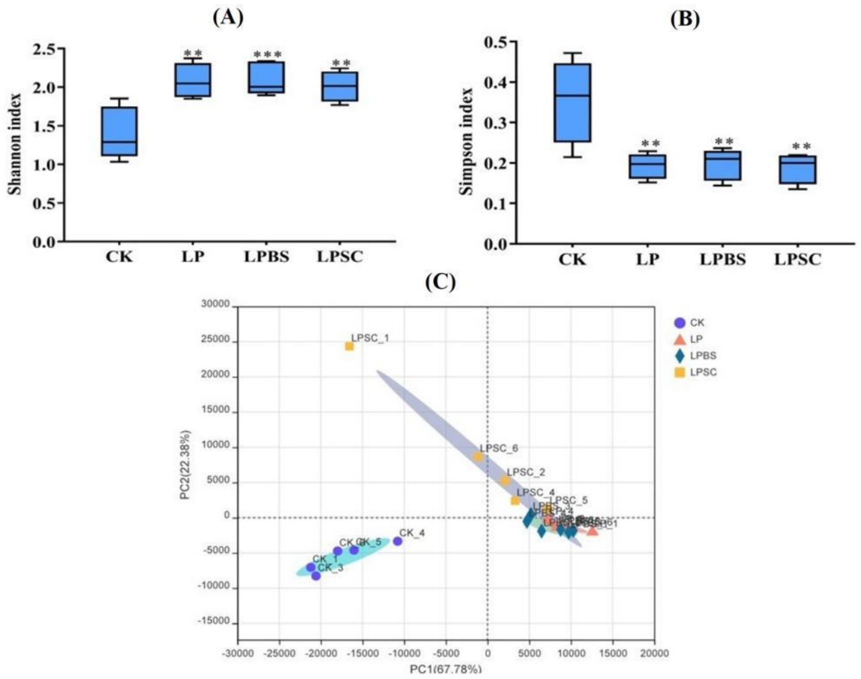

Supplementary Figure 2. Alpha (Shannon and Simpson indices) and beta (PCA) diversities analysis of fungal community of whole plant soybean silage after 60 days of ensiling. A for Shannon index; B for Simpson index; C for PCA. CK, sterilized water; LP, *Lactiplantibacillus plantarum* B90; LPBS, LP combined with *Bacillus subtilis* C5B1; LPSC, LP combined with *Saccharomyces cerevisiae* LO-1

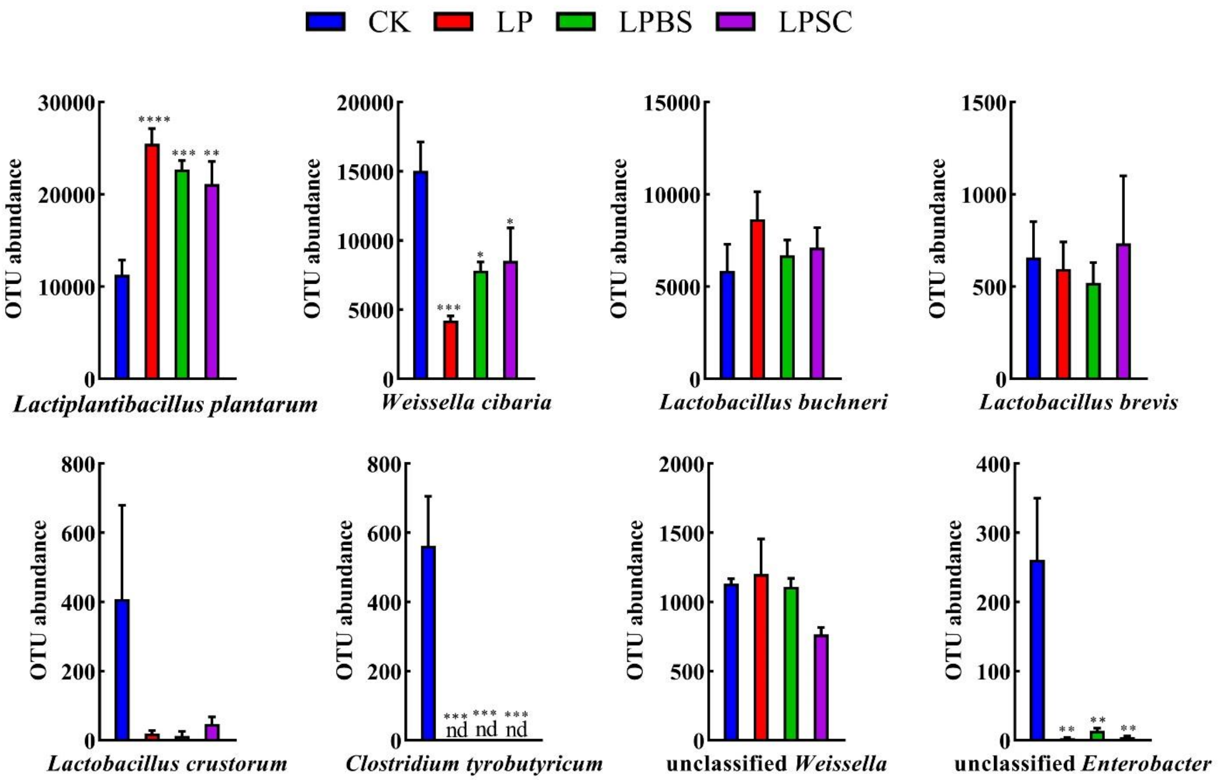

Supplementary Figure 3. The relative abundances of main bacterial species on OTU level of whole plant soybean silage after 60 days of ensiling. CK, sterilized water; LP, *Lactiplantibacillus plantarum* B90; LPBS, LP combined with *Bacillus subtilis* C5B1; LPSC, LP combined with *Saccharomyces cerevisiae* LO-1.

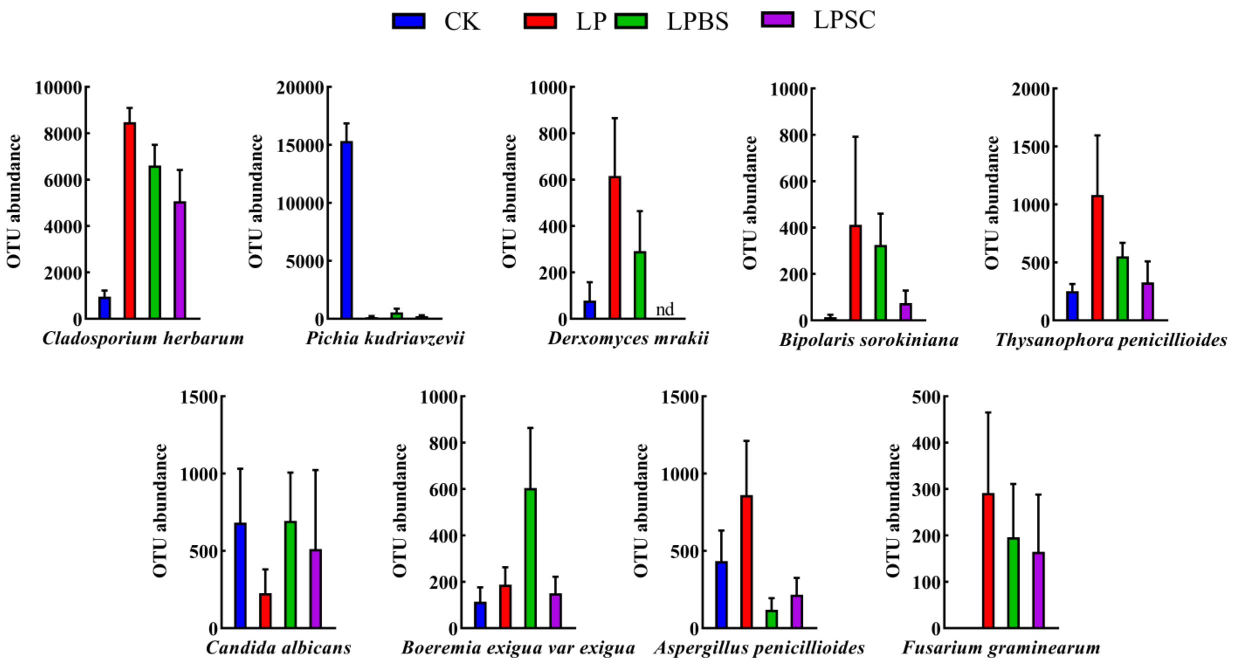

Supplementary Figure 4. The relative abundances of main fungal species on OTU level of whole plant soybean silage after 60 days of ensiling. CK, sterilized water; LP, *Lactiplantibacillus plantarum* B90; LPBS, LP combined with *Bacillus subtilis* C5B1; LPSC, LP combined with *Saccharomyces cerevisiae* LO-1.

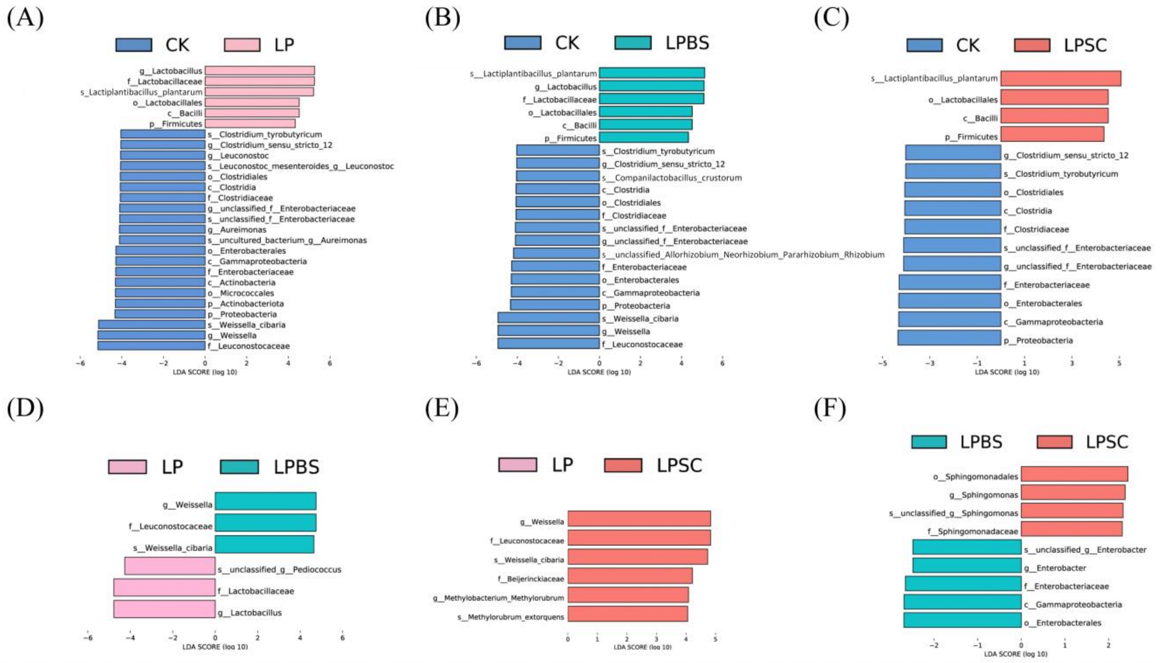

Supplementary Figure 5. Linear discriminant analysis effect sizes (LEfSe) were used to assess differences in bacterial communities between CK and inoculant groups of WPS silage (LDA scores > 4.0; LPBS vs LPSC, LDA score > 2.0). The length of the histogram represents the LDA score of different species. CK, sterilized water; LP, *Lactiplantibacillus plantarum* B90; LPBS, LP combined with *Bacillus subtilis* C5B1; LPSC, LP combined with *Saccharomyces cerevisiae* LO-1

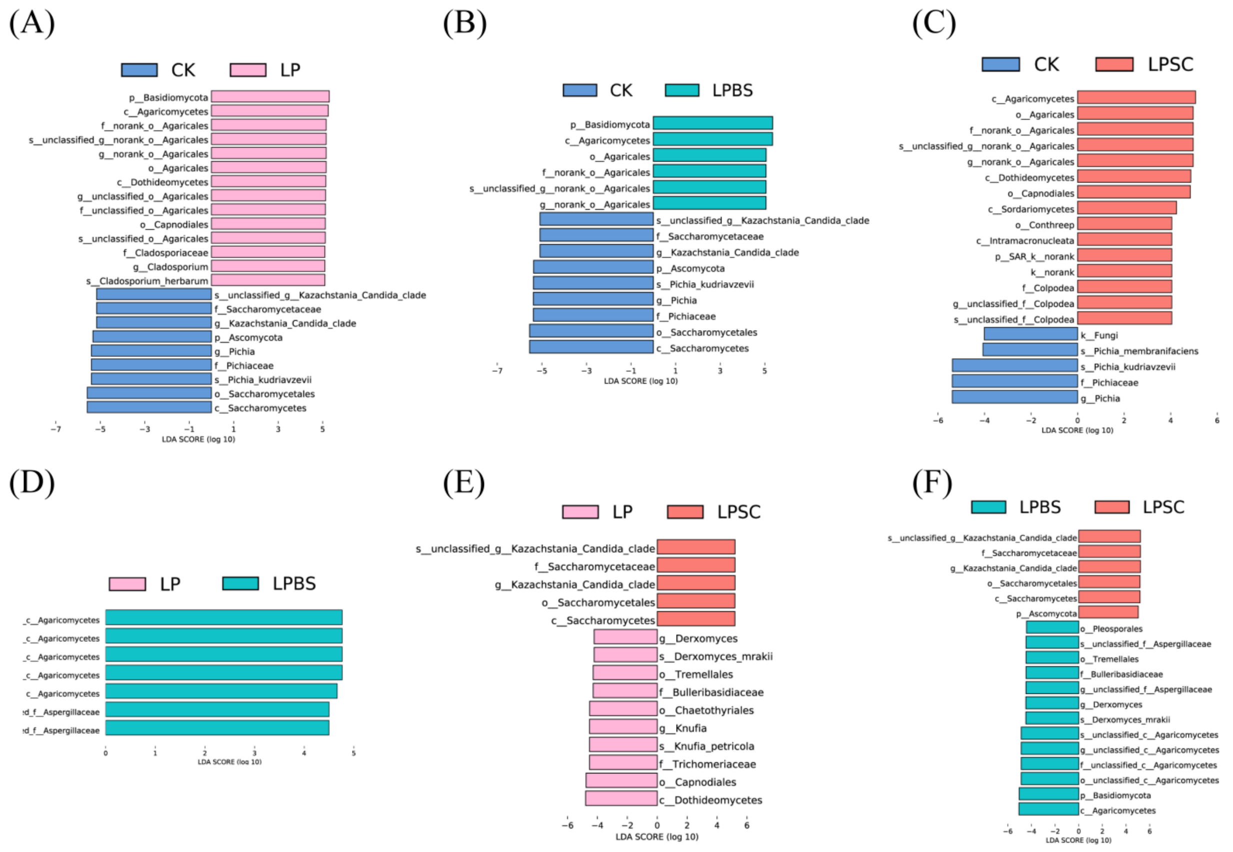

Supplementary Figure 6. Linear discriminant analysis effect sizes (LEfSe) were used to assess differences in fungal communities between CK and inoculant groups of WPS silage (LDA scores > 4.0; CK vs LP, CK vs LPBS, LDA scores > 5.0). The length of the histogram represents the LDA score of different species. CK, sterilized water; LP, *Lactiplantibacillus plantarum* B90; LPBS, LP combined with *Bacillus subtilis* C5B1; LPSC, LP combined with *Saccharomyces cerevisiae* LO-1.
